# Investigating past range dynamics for a weed of cultivation, *Silene vulgaris*

**DOI:** 10.1002/ece3.2250

**Published:** 2016-06-16

**Authors:** Megan E. Sebasky, Stephen R. Keller, Douglas R. Taylor

**Affiliations:** ^1^ Department of Biology University of Virginia Charlottesville Virginia; ^2^ Department of Plant Biology University of Vermont Burlington Vermont

**Keywords:** Glacial refugia, last glacial maximum, MaxEnt, phylogeography, postglacial expansion, *Silene vulgaris*, species distribution model

## Abstract

Since the last glacial maximum (LGM), many plant and animal taxa have expanded their ranges by migration from glacial refugia. Weeds of cultivation may have followed this trend or spread globally following the expansion of agriculture or ruderal habitats associated with human‐mediated disturbance. We tested whether the range expansion of the weed *Silene vulgaris* across Europe fit the classical model of postglacial expansion from southern refugia, or followed known routes of the expansion of human agricultural practices. We used species distribution modeling to predict spatial patterns of postglacial expansion and contrasted these with the patterns of human agricultural expansion. A population genetic analysis using microsatellite loci was then used to test which scenario was better supported by spatial patterns of genetic diversity and structure. Genetic diversity was highest in southern Europe and declined with increasing latitude. Locations of ancestral demes from genetic cluster analysis were consistent with areas of predicted refugia. Species distribution models showed the most suitable habitat in the LGM on the southern coasts of Europe. These results support the typical postglacial northward colonization from southern refugia while refuting the east‐to‐west agricultural spread as the main mode of expansion for *S. vulgaris*. We know that *S. vulgaris* has recently colonized many regions (including North America and other continents) through human‐mediated dispersal, but there is no evidence for a direct link between the Neolithic expansion of agriculture and current patterns of genetic diversity of *S. vulgaris* in Europe. Therefore, the history of range expansion of *S. vulgaris* likely began with postglacial expansion after the LGM, followed by more recent global dispersal by humans.

## Introduction

Determining the limits of species geographic ranges and their movements over time has been a fundamental goal for ecology (MacArthur 1972) and evolutionary biology (Excoffier et al. 2009). Large‐scale climate change is known to be important for determining species distribution shifts (Davis and Shaw 2001; McCarty 2001; Walther et al. 2002). Historical climatic oscillations associated with glacial ice ages influenced the cyclical expansion and contraction of many species ranges. Evidence for the “expansion–contraction” model (Provan and Bennett 2008) has been observed in the phylogeographic patterns of genetic diversity for terrestrial flora and fauna in Europe and North America (reviewed in Hewitt (2004) and Schmitt (2007)). However, multiple historical and environmental factors may structure phylogeographic patterns of genetic diversity. For example, plastic species that can survive in a wide range of climates may be better able to endure environmental changes in situ, and thus not adhere to the typical “expansion–contraction” model.

Weeds of cultivation are dispersed widely and unintentionally by human agricultural practices and will thrive in disturbed areas across a broad range of climatic environments. For a weed of cultivation, human‐mediated dispersal during the expansion of agriculture may be the dominant process shaping the spatial pattern of its diversity, rather than the expansion–contraction model of postglacial expansion following the last glacial maximum (LGM, ca. 20,000 years ago; Balfourier et al. 2000). Previous phylogeographic studies of postglacial expansion have mainly focused on animal and tree species as opposed to weedy herbaceous plants (Sharbel et al. 2000; François et al. 2008). The few phylogeographic studies of widespread, weedy plant species find different patterns of diversity than expected from models of postglacial expansion, most likely due to recent human‐mediated dispersal (Tyler 2002; Prentice et al. 2008; Jiménez‐Mejías et al. 2012). A recent study of two weeds of cultivation, *Lolium perenne* and *L. rigidum*, in Europe found demes (i.e., ancestral genetic clusters) whose distributions were correlated with historical agricultural routes (Balfourier et al. 2000), while another study found support for postglacial expansion of multiple demes structured between different putative glacial refugia in *Arabidopsis thaliana* (Beck et al. 2008).

Testing alternative hypotheses such as postglacial expansion v. human‐mediated agricultural spread would benefit from combining genetic data with spatial models to refine predictions about habitat–diversity relationships under each scenario. Recently, phylogeographic approaches have been used in concert with species distribution modeling (SDM) to reconstruct past species range dynamics, especially for taxa lacking fossil records. SDMs can also be used to hypothesize about the location of glacial refugia, augmenting findings based on population genetic data. SDMs use current species locations and environmental variables to fit habitat suitability models. These models can be trained on current climatic data and projected onto different climatic datasets to predict species ranges in different regions or periods of time. For past range dynamics, current climate suitability models can be projected onto the reconstructed climate data for the LGM to determine putative locations of glacial refugia (Kozak et al. 2008).

Species distribution modelings have many assumptions and limitations (e.g., see Araújo and New 2007; Diniz‐Filho et al. 2009) as well as high uncertainty when projecting in space and time (Elith and Leathwick 2009; Nogués‐Bravo 2009). However, combining SDMs with population genetic data can enable more robust assessments of historical range dynamics (Waltari et al. 2007; Schorr et al. 2012, 2013; Waltari and Hickerson 2012). Population genetic analyses allow the identification of glacial refugia areas with high genetic diversity where populations are hypothesized to be able to survive through the LGM (Hewitt 2000; Petit et al. 2003). This signature of high genetic diversity in older, refugial populations with low diversity on the outskirts of the range would support the “leading edge” model of range expansion in which only a subset of individuals (e.g., founders) at the expansion front establish and come to dominate the population (Hewitt 1996; Bialozyt et al. 2006).

In Europe, postglacial expansion primarily occurred by species spreading northward from southern glacial refugia on Europe's Mediterranean peninsulas (Hewitt 2000). In contrast, the more recent agricultural spread by humans originated in the Middle East and spread westward into Europe (Ammerman and Cavalli‐Sforza 1971; Pinhasi et al. 2005). Therefore, the competing hypotheses for expansion can be tested by comparing estimated gradients in genetic diversity with latitude and longitude. Genetic structure analyses can also help identify refugial populations through geographic clustering of demes. In Europe, it is common to see up to three demes whose descendants are spread latitudinally from refugia in the three southern peninsulas of Iberia, Italy, and the Balkans (Taberlet et al. 1998; Hewitt 2004; Provan and Bennett 2008). Similarly, demes can be identified for weeds of cultivation that cluster based on the route of westward agricultural spread (Balfourier et al. 2000). Population genetic methods can be quite useful in determining past range dynamics, but historical processes can be difficult to disentangle from more recent events, especially in the case of contemporary admixture (Petit et al. 2003).

In this study, we used the combined application of phylogeographic analysis and SDM to examine whether the weedy plant *Silene vulgaris* (Moench) Garcke has become widespread during historical postglacial expansion or through more recent agricultural expansion. Past genetic studies of *S. vulgaris* have found phylogeographic signatures of ancestral demes dispersed throughout Europe, making it difficult to predict past range dynamics (Taylor and Keller 2007; Keller and Taylor 2010; Keller et al. 2014). Here, we add to the data from Keller et al. (2014) by analyzing microsatellite data for additional samples with a greater representation of eastern and southern European populations from classic refugial areas (Iberia, Italy, Balkans). This more robust sampling of populations across the European range allowed us to test two competing hypotheses: (1) the range expansion in *S. vulgaris* followed typical post‐glacial expansion routes northward from southern refugia since the LGM or (2) followed the spread of agriculture, westward from the Middle East as humans created disturbed land and transported seeds.

## Materials and Methods

### Phylogeography

#### Population samples and genotyping

**Figure 1:**
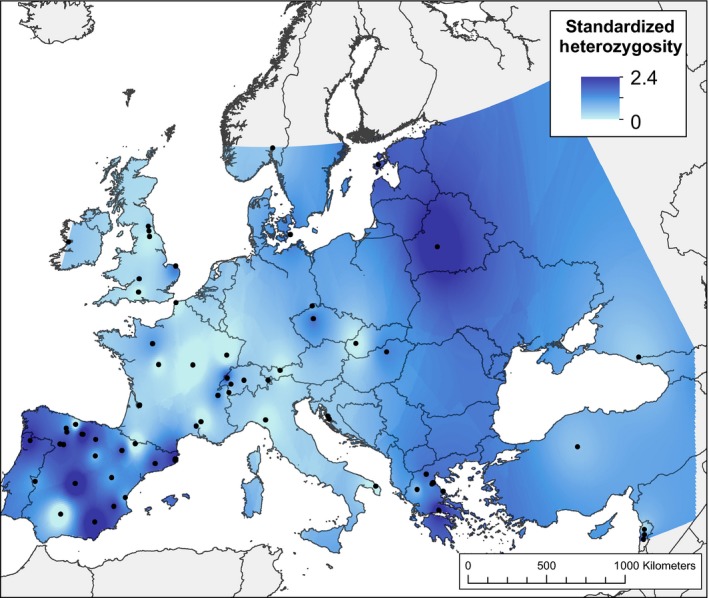
Standardized heterozygosity calculated for *Silene vulgaris* individuals in Europe and interpolated using the inverse distance weighting method (IDW) in ArcGIS 10.1. Warmer colors show higher genetic diversity, while cooler colors show lower genetic diversity. Dark blue circles show populations used in the analysis.

We sampled 167 individuals from 73 populations across the native range of *S. vulgaris* in Europe, with one to 10 individuals sampled per population. Samples were collected as seeds from maternal families or as leaf tissue dried on silica gel (Keller and Taylor 2010). Genomic DNA was extracted from leaf tissue using Qiagen DNeasy Plant Mini Kit (Qiagen, Valencia, CA). We genotyped 10 of the 15 markers used in Keller et al. (2014) and derived from *S. latiolia* as described by Moccia et al. (2009; Table 1). Microsatellite amplification and fragment analysis were performed as described in Keller et al. (2014). Genotyping and binning of the 167 new samples as well as an additional 79 samples from Keller et al. (2014) was performed using GeneMarker 2.6.2 (Softgenetics, LLC, State College, PA). One marker, SL_eSSR17, was removed from the analysis due to peaks of varying sizes inconsistent with the known number of repeats. Individuals with missing data at more than three loci were removed, giving a total of 191 individuals in 76 populations genotyped at nine loci (Fig. 1).

**Table 1:** Microsatellite markers and associated genetic diversity metrics for *Silene vulgaris* populations in Europe

Locus	Indiv. scored	No. alleles	Eff. alleles	*H*_O_	*H*_S_	*H*_t_	*H*'_t_	*G*_is_
SL_eSSR01	123	5	1.387	0.274	0.579	0.558	0.557	0.526
SL_eSSR03	137	12	1.42	0.323	0.584	0.816	0.819	0.447
SL_eSSR04	182	7	1.233	0.269	0.28	0.49	0.493	0.038
SL_eSSR05	172	7	1.414	0.414	0.421	0.567	0.569	0.017
SL_eSSR012	168	17	1.859	0.613	0.744	0.903	0.906	0.176
SL_eSSR016	184	9	1.455	0.408	0.478	0.632	0.634	0.145
SL_eSSR20	160	5	1.232	0.166	0.38	0.52	0.522	0.562
SL_eSSR22	174	6	1.118	0.106	0.203	0.226	0.227	0.48
SL_eSSR28	154	9	1.39	0.381	0.468	0.622	0.624	0.187
Overall		8.556	1.39	0.328	0.46	0.593	0.595	0.286

*H*_O_, Observed heterozygosity; *H*_S_, heterozygosity within populations; *H*_t_, total heterozygosity; *H*'_t_, corrected total heterozygosity; *G*_is_, inbreeding coefficient.

#### Estimation of genetic diversity

Due to small sample sizes within most populations, we estimated metrics of genetic diversity at the individual level and compared these to diversity estimates for populations of *n* > 1. Multilocus heterozygosity was calculated for each individual using the standardized heterozygosity metric within the Rhh package (Alho et al. 2010), which is calculated as the proportion of heterozygous‐typed loci/mean heterozygosity of typed loci (Coltman et al. 1999). Observed (*H*_o_) heterozygosity estimates for the 45 populations of *n* > 1 were calculated in GenoDive (Meirmans and Van Tienderen 2004). To assess the spatial distribution of genetic diversity, the two metrics were interpolated across the study area using the inverse distance weighting method through the SPATIAL ANALYST extension in ArcGIS 10.1 (ESRI, Redlands, CA). The similarity of the results for the two metrics (Appendix S1) supported the use of standardized heterozygosity going forward.

#### Phylogeographic structure

To assess geographic patterns of genetic ancestry in different historically isolated groups, we used Bayesian clustering to assign multilocus genotypes into clusters using the program STRUCTURE version 2.3 (Pritchard et al. 2000). We performed 10 independent runs for each *K* (1–10) with the default program settings and 1,000,000 MCMC iterations after a burn‐in period of 500,000 iterations. The optimal number of clusters (*K*) was determined based on the (Evanno et al. 2005) method implemented in STRUCTURE HARVESTER (Earl and vonHoldt 2012). We used CLUMPP (Jakobsson and Rosenberg 2007) to align the ancestry coefficients (*Q*‐values) across the 10 replicates. Results were visualized using pie charts of population‐averaged ancestry coefficients and mapped using ArcGIS 10.1 (ESRI, Redlands, CA).

### Species distribution modeling

To identify locations of putative glacial refugia, we used MaxEnt to create SDMs to predict areas of suitable habitat for *S. vulgaris* during the LGM based on its current environmental niche. To evaluate the plausibility of using climate‐only data when hindcasting models to the LGM, we first assessed whether the current niche of *S. vulgaris* depends more on climate than other factors that may influence its distribution. In the “climate plus environment” model (model C + E), we created the SDM using both climate as well as present‐day environmental variables that are not available for the LGM. For the “climate‐only” model (model C), we created a second SDM that only included climate variables that were also available for projecting into the LGM.

#### Occurrence data

For both the current and LGM models, we trained the SDMs based on occurrence data from D. R. Taylor's seed‐collection database as well as records from the Global Biodiversity Information Facility (GBIF; http://www.gbif.org). Our initial dataset included 219 seed collection locations and 14,238 post‐1950 records available from GBIF. The reported and observed spatial resolution (where available) for the majority of these points was 10 km^2^ or less. However, inspection of the GBIF dataset revealed obvious reporting biases among different locations, which could result in spatial autocorrelation. Overall, GBIF occurrence locations were biased toward western Europe, with many points covering western Europe and extremely few in eastern Europe, where *S. vulgaris* is also widespread based on the Atlas Florae Europeae (Jalas and Suominen 1986). This was most extreme in the United Kingdom, where reported occurrences covered nearly the entire country. To reduce spatial bias and the potential for spatial autocorrelation, we reduced the dataset to one point per 10 km^2^ grid cell using ArcGIS 10.1, thus matching the resolution of the environmental data as well as the resolution of the majority of GBIF records. For the UK, we also excluded GBIF records and instead used updated records obtained directly from the main source of GBIF data in that region, the Botanical Society of Britain and Ireland. The resulting occurrence dataset for model training included 3173 points. We further accounted for the spatial bias in the dataset using a bias grid during modeling.

#### Present‐day environmental data

For the present‐day and LGM SDMs, we obtained data for 19 bioclimatic variables from the WorldClim database (Hijmans et al. 2005). These variables are derivatives of temperature and precipitation patterns that may better reflect the aspects of climate driving species distributions than raw recorded measurements. We downloaded the data at a resolution of 2.5 arc minutes and resampled to 10 km^2^ resolution in ArcGIS 10.1 using the bilinear resampling technique suitable for continuous data. To account for collinearity among the 19 bioclimatic variables, we ran a principal components analysis (PROC PRINCOMP; SAS version 9.4, SAS Institute 2012). The first two principal components accounted for 74.3% of the variation in the 19 bioclimatic variables (Appendix S2 in Supporting information). In model C + E, we also included data on soil type, land use, and human influence, as these variables could be important for describing the niche of a widespread weed of cultivation. For soil type, we used multiple datasets from the European Soil Database version 2 at 10 km^2^ resolution: full soil code of the soil typological unit (STU) from the World Reference Base (WRB) for Soil Resources (WRB‐FULL), dominant parent material of the STU (PAR‐MAT‐DOM), full soil code of the STU from the 1974 (modified CEC 1985) FAO‐UNESCO Soil Legend (FAO85‐FULL), and dominant land use (USE‐DOM). We also included a more detailed land‐use dataset from the European Environment Agency, the Corine Land Cover 2006 database, version 16, downloaded at 250‐m resolution (Copyright ^©^ European Environment Agency). For another measure of human disturbance, we used the Last of the Wild version 2 Human Influence Index dataset at 1‐km resolution (Wildlife Conservation Society ‐ WCS and Center for International Earth Science Information Network–CIESIN–Columbia University 2005). The Human Influence Index was created from data layers including population density, land use and infrastructure, and human access (coastlines, roads, railroads, rivers). All datasets were resampled to 10‐km^2^ resolution. Because some datasets did not cover the entire study area, we extracted all data to the smallest extent of the input grids. A second principal component analysis was performed for these data to account for collinearity among the soil, land use, and human influence datasets (PROC PRINQUAL; SAS version 9.4, SAS Institute 2012). The first two principal components explain 92.8% of the variation in the datasets (Appendix S2). The first two bioclimatic and two environment‐based principal components were used as environmental variables for the C + E model (Table 2).

**Table 2:** Environmental variables used in the current and last glacial maximum (LGM) species distribution models (SDMs) for *Silene vulgaris* in Europe

Current SDM	LGM (CCSM and MIROC) SDMs
Bioclim PC1	Max. temp. warmest month
Bioclim PC2	Temp. annual range
Landcover C1	Mean temp. wettest quarter
Landcover C2	Mean temp. coldest quarter
	Precip. driest month
	Precip. seasonality
	Precip. coldest quarter

#### LGM environmental data

The 19 bioclimatic variables for current conditions were used to train the LGM MaxEnt model. In the case of the LGM model, collinearity was not addressed using principal components analysis because it assumes constancy in the correlation structure among different aspects of climate, which is likely to be false. To confirm this, principal components analyses were run on both the current climate and LGM climate data, and the relationships between variables differed considerably. To address collinearity issues in the LGM model, we ran a correlation analysis in ENMTools (Warren et al. 2010) and reduced the dataset to seven bioclimatic variables (Table 2) with correlation coefficients of <0.7. From each pair of variables with *r *>* *0.7, one variable was kept in the model based on variable importance and degree of extrapolation in the LGM in initial model runs. The degree of extrapolation was determined by viewing the most dissimilar variable (MoD) output maps provided by MaxEnt (Elith et al. 2010). Climatic data for the LGM were obtained from the WorldClim database (Hijmans et al. 2005) at 2.5‐arc‐min resolution for both the available datasets based on two general circulation models (GCMs): Community Climate System Model (CCSM version 3; Collins et al. 2006) and Model for Interdisciplinary Research on Climate (MIROC version 3.2; (Hasumi and Emori 2004), and resampled to 10‐km^2^ resolution. Independent models projecting *S. vulgaris* distributions in the LGM were performed using both LGM climate datasets based on different GCMs for comparison, as neither GCM is known to be more accurate.

#### SDM procedure

We utilized the machine learning method based on maximum entropy implemented in the program MaxEnt 3.3.3k (Phillips et al. 2006) to assess the current environmental niche of *S. vulgaris* and hindcast its distribution during the LGM. We created a sample bias grid using SDMToolbox version 1.0b (Brown 2014) using the Gaussian kernel density of sampling localities. The sampling bias distance to create the grid (20 km) was chosen to minimize the influence of very high sampling density in parts of western Europe and give a projected current distribution that corresponds more closely with observations from the Atlas Florae Europeae than the model without the bias grid. We ran MaxEnt for 10 replicates using default program settings with the addition of the bias grid and averaged the predictions across replicates. For the LGM models, multivariate environmental similarity surface (MESS) and MoD maps (Elith et al. 2010) were evaluated to assess the extent that models may be affected by nonanalog climate conditions when projecting from current conditions to the LGM. The MIROC model predicted substantial suitable habitat up to 50°N (Appendix S3), yet it is known from climate reconstructions that the LGM ice sheet extended south down to about 52°N and permafrost covered most areas south to 47°N (Hewitt 2004). The MIROC model was therefore was removed from the analysis, and instead, the CCSM model was used because the predictions were plausible given the extent of glaciation.

#### Correlation between genetic diversity and spatial data

We used Spearman's rank correlation in JMP 9 (SAS Institute Inc., Cary, NC, version 9.4 for Windows) to test for associations between standardized heterozygosity and three predictors: latitude, longitude, and LGM climate suitability. Based on our two competing hypotheses, we predicted a significant correlation between diversity and longitude would support the agricultural expansion hypothesis, whereas a significant correlation between diversity and latitude or LGM suitability would support the postglacial expansion hypothesis.

## Results

### Genetic diversity

The spatial distribution of individual standardized heterozygosity revealed complex latitudinal and longitudinal patterns in the genetic diversity of *S. vulgaris* across Europe (Fig. 1). In western Europe, heterozygosity was highest in Spain and decreased northeastward to low levels from Italy to the UK and Ireland. In eastern Europe, diversity was highest in Greece and Belarus, and decreased to the east of these countries (Fig. 1). These patterns manifested in four latitudinal groups based on similar diversity levels: (1) high diversity in Spain, (2) low diversity from Italy to Ireland, (3) high diversity from Greece to Estonia and northwestern Russia, and (4) low diversity from Lebanon and Turkey to southwestern Russia.

### Genetic structure

**Figure 2:**
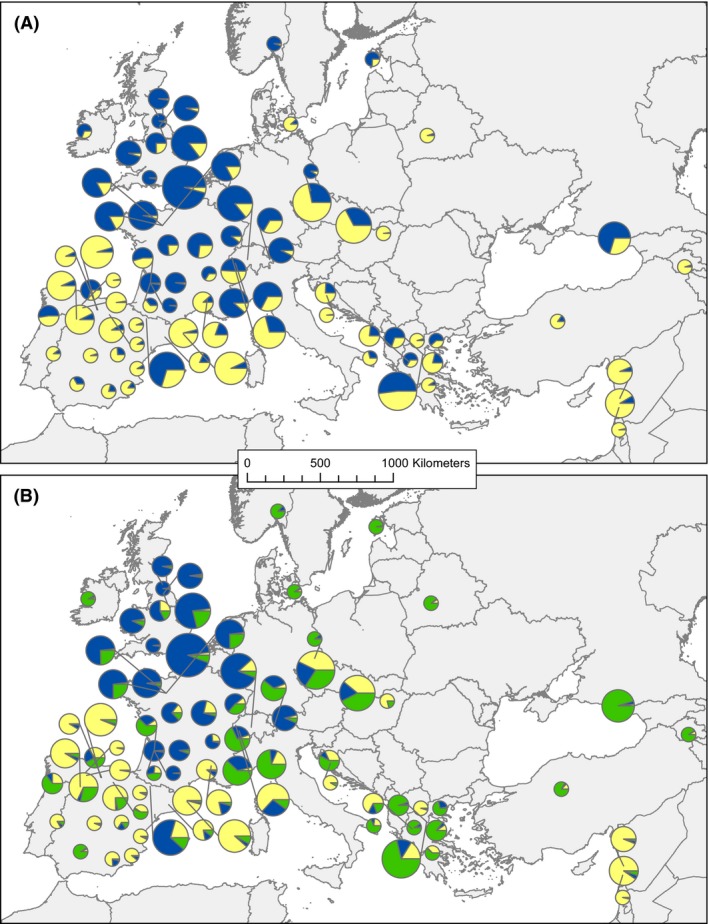
Ancestry assignment from STRUCTURE models for *Silene vulgaris* populations in Europe. (A) Map showing pie charts of population‐averaged ancestry assignment for *K* = 2. Size of circles indicates sample size of each population. (B) Same as (A) for *K* = 3.

Bayesian clustering using STRUCTURE described an optimal model of *K* = 2 clusters based on the Δ*K* method (Evanno et al. 2005). In western Europe, the two clusters roughly separated into a northern cluster in France and the U.K. (blue) and another more southern cluster most frequent in the Iberian Peninsula (Fig. 2A). In eastern Europe, many populations were of mixed ancestry, although there were several populations in the Middle East that had a high proportion of ancestry in the yellow cluster. At *K* = 3, populations in eastern Europe showed high posterior probability for a third genetic cluster, but it was also present in many western populations (Fig. 2B). Overall, the analysis roughly distinguished populations from Iberia and the Middle East (yellow), northwestern Europe (blue), and eastern Europe (green), although admixture was also common (Fig. 2B).

### SDM for current and LGM conditions

The MaxEnt model based on both climate and other environmental variables (model C + E) was an adequate fit based on test AUC (0.705, training 0.712) and the known current distribution of the species. An AUC value above 0.7 indicates “fair” model performance (Swets 1988; Araújo et al. 2005), and the maximum achievable AUC is lower for a widespread species (Phillips et al. 2006). Using a bias grid of Gaussian kernel density at 20 km resulted in a prediction of current distribution consistent with field observations and the Atlas Florae Europeae (Jalas and Suominen 1986) (Appendix S4). All variable importance metrics showed both climatic principal components having an overwhelmingly large effect on the distribution of *S. vulgaris* when compared to the land‐use variables (Table 3). A similar result was found using a model with all of the original variables before principal components analyses (data not shown). The climate‐only model (model C) with the reduced set of bioclimatic variables returned a slightly higher test AUC (0.772, training AUC 0.780) than model C + E and a predicted current distribution consistent with field observations and the Atlas Florae Europeae (Appendix S4). The increased fit of model C over model C + E lends support to predicting distributions back into the LGM, for which only climate data are reconstructed.

**Table 3:** Percent contribution and permutation importance values for each environmental variable used in the MaxEnt model for the prediction of current distribution of *Silene vulgaris* in Europe. Each environmental variable is a principal component axis summarizing multiple datasets. Bioclim PC1 and PC2 are the first two principal components of the 19 bioclimatic variables. Landcover C1 and C2 are the two components representing soil type, land cover, and human influence metrics

Variable	Percent contribution	Permutation importance
Bioclim PC2	70.2	60.5
Bioclim PC1	27.5	32.1
Landcover C1	1.4	6
Landcover C2	1	1.4

**Figure 3:**
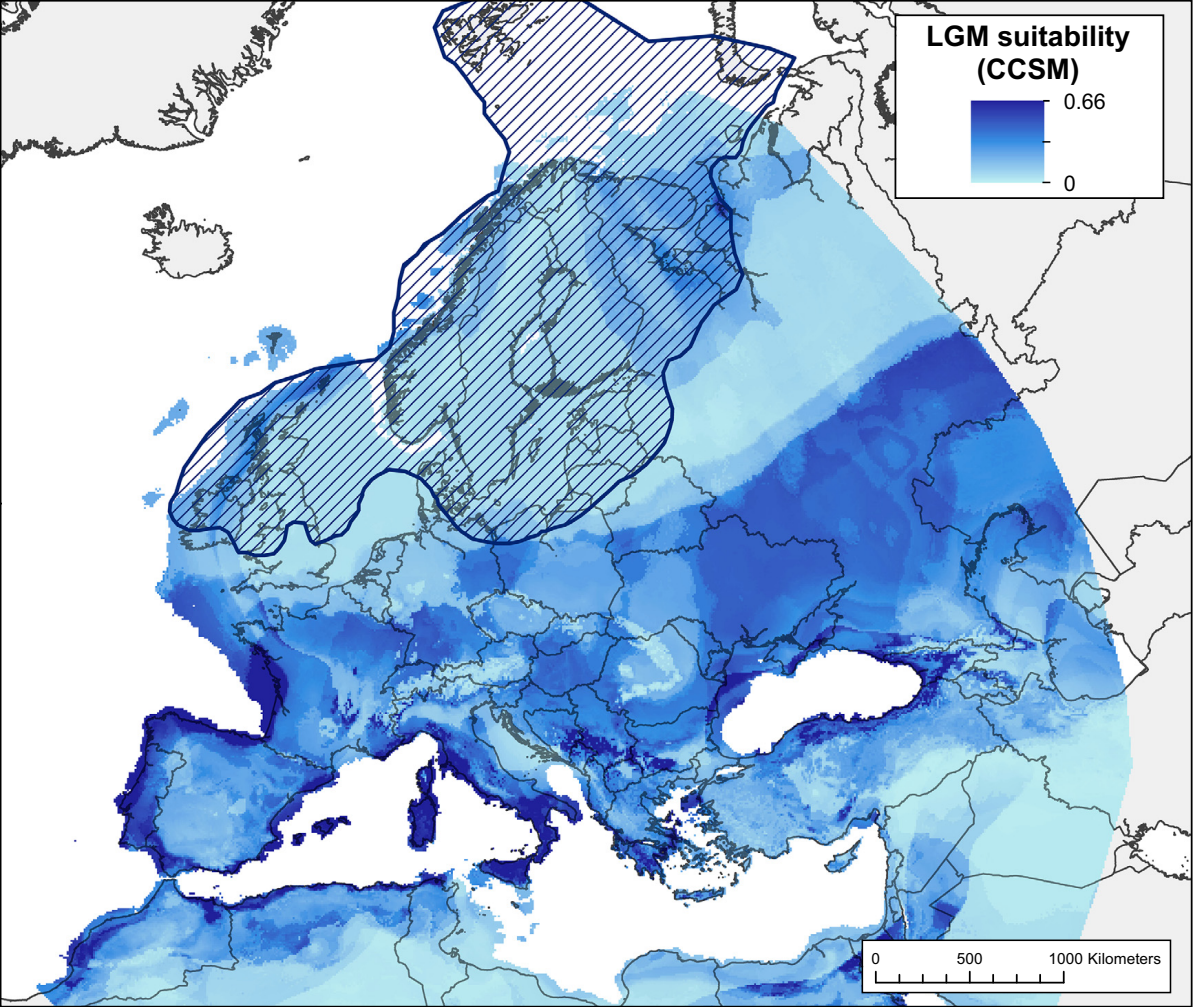
Predicted climate suitability for *Silene vulgaris* in Europe during the last glacial maximum (LGM) based on the community climate system model (CCSM) climate scenario. The hashed blue area shows a generalized extent of the ice sheets during the LGM (Svendsen et al. 2004).

The LGM prediction from the CCSM model showed moderately suitable habitat in most of the regions of Europe not covered by Eurasian ice sheets (Fig. 3) with higher suitability in southern Europe along the coasts. Notably, the model predicted high values for suitable habitat on all three European peninsulas predicted to be major glacial refugia for temperate plant and animal species.

A common concern with the SDM approach is the extent that predictions of suitable habitat can be biased when extrapolating into environmental space outside the known area of occurrence. For *S. vulgaris*, the MESS map (Appendix S5) showed that the variables in our model were only extrapolated from their training range into northern Europe, predominantly in the location of the Eurasian ice sheet, above 52°N. Even with the extrapolation, the MaxEnt models correctly predicted very low suitability in this area, except farther north near Finland and Russia where there was the largest degree of extrapolation.

### Spatial patterns of genetic diversity

Spearman's rank correlation showed that standardized heterozygosity significantly declined with latitude (Table 4). Genetic diversity was not associated with any other spatial predictors; notably, there was no evidence for increased genetic diversity toward the origin of agricultural expansion in the east. These results persisted when admixed individuals (0.25 < *Q* < 0.75 for *K* = 2) were removed from the analysis. In addition, when admixture is removed, there was a significant (*P* = 0.034) positive relationship between LGM suitability and heterozygosity, consistent with high genetic diversity in putative southern refugia.

**Table 4:** Correlation analysis to assess the relationship between genetic diversity (standardized heterozygosity) of *Silene vulgaris* individuals in Europe and four variables: geographic location (latitude, longitude), and last glacial maximum climate suitability based on the Community Climate System Model (CCSM) general circulation model. Bold indicates significant *P*‐values after Bonferroni correction

Variable	Variable	Spearman's *ρ*	Prob > |*ρ*|
Latitude	Heterozygosity	−0.3478	**<0.0001**
Longitude	Heterozygosity	−0.0919	0.2061
Suitability (CCSM)	Heterozygosity	0.0791	0.277
Suitability (CCSM)	Latitude	−0.4692	**<0.0001**

## Discussion

We used population genetic analysis of microsatellite diversity and SDM for *S. vulgaris* throughout Europe in order to reconstruct past range dynamics and test two competing hypotheses for range expansion: postglacial expansion from southern refugia versus westward agricultural expansion. The results support the hypothesis of postglacial expansion from southern refugia and are not consistent with predictions of agricultural spread from the east. These findings are in contrast with the only other study that has contrasted the same hypotheses for two weeds of cultivation, *L. perenne* and *L. rigidum* (Balfourier et al. 2000). Their finding was based on detecting genetic structure among populations along longitudinal trade routes; however, their study did not estimate clines in genetic diversity that would point to centers of origin and range expansion. Our findings are similar to those found in a recent study of *A. thaliana* (Beck et al. 2008). Evidence of postglacial expansion is still apparent in the genomes of both *A. thaliana* and *S. vulgaris* even though phylogeographic patterns may become obscured by ongoing dispersal and admixture, either as a natural product of secondary contact between different refugia during expansion (Petit et al. 2003) or as a result of more recent human‐mediated dispersal (Wilson et al. 2009).

### Genetic diversity trends support postglacial expansion

Genetic diversity of *S. vulgaris* declined along a latitudinal gradient. High genetic diversity in Spain and Greece supports the existence of glacial refugia in those areas, consistent with known refugia for temperate plant and animal species on the southern European peninsulas. The Italian peninsula also commonly served as a refugium for many species during the LGM (Taberlet et al. 1998; Hewitt 1999). Although the spatial patterns of genetic diversity suggest the existence of refugia on only two of the three southern peninsulas, our sampling on the Italian peninsula (one individual from one population in southern Italy) does not allow us to confirm or refute an Italian refugium for *S. vulgaris*. The significant negative correlation between heterozygosity and latitude (i.e., lower diversity at northern latitudes) is consistent with range expansion out of southern refugia, in which founder effects (genetic drift during population establishment) reduced diversity along the pathways of expansion. Climate suitability in the LGM model was also significantly negatively correlated with latitude, providing further evidence that *S. vulgaris* likely formed southern refugial populations. Taken together, the reconstruction of the climate during the LGM and current patterns of genetic diversity are consistent with expectations of a postglacial expansion from southern European refugia. Further, when admixed individuals were removed from the analysis, there was a significant positive correlation between LGM suitability and heterozygosity. By contrast, there was not a significant correlation between heterozygosity and longitude, and the sign of this correlation was negative, contrary to hypotheses of *S. vulgaris* migrating westward during agricultural expansion. There was also low habitat suitability in the Middle East in the current and LGM models, further supporting that the center of origin was not located in this area. These findings exemplify the benefit of combining genetic, spatial, and suitability data to understand range dynamics and discriminate between competing hypotheses for the historical spread of weedy plants.

### Genetic structure and location of potential glacial refugia

In western Europe, west of Germany, and Italy, the STRUCTURE analysis found evidence for two demes consistent with expectations for divergent groups of descendants from different glacial refugia (Taberlet et al. 1998; Hewitt 2004; Provan and Bennett 2008). Our results suggest one refugia was likely located on the Iberian Peninsula with the Pyrenees mountains acting as a barrier to dispersal, a pattern seen in other species as well (Taberlet et al. 1998; Hewitt 2000; Schmitt 2007). The origin of the second western European deme is unclear. The LGM reconstructions supported suitable habitat in coastal France or Italy suggesting a possible refugium in one of these regions; however, there was no clear area of higher genetic diversity in either area, and our sampling in Italy was too sparse to robustly detect a refugial location within this region.

The population genetics of *S. vulgaris* in eastern Europe are not straightforward. Many individuals in eastern Europe, especially those in Lebanon, were genetically similar to individuals on the opposite edge of the species range on the Iberian Peninsula, and a second cluster originating in France/Italy separated these two regions. This pattern could have resulted from recent human‐mediated dispersal, perhaps via trade routes along the North African coast (Balfourier et al. 2000). However, a similar genetic clustering pattern was observed in a recent study of the European wild boar (Vilaça et al. 2014), which would not likely follow these same trade routes. (Vilaça et al. 2014) proposed a scenario during the last interglacial period, where Iberian and eastern European populations could have traveled northward and become panmictic, with populations in France and Italy remaining isolated. This seems unlikely for *S. vulgaris* because the Pyrenees seem to be a barrier to dispersal that would have remained as such during past interglacial periods. However, Iberian and Lebanon populations may have originated from panmictic populations in northern Africa.

Another possible explanation for the unexpected similarity between eastern and western regions of the Mediterranean is that there are three refugial groups originating in the three southern peninsulas, but STRUCTURE was unable to separate a third cluster based on the data. The STRUCTURE analysis showed moderate support for *K* = 3, which supports the differentiation between Iberia and eastern populations, except for those in Lebanon. With more data from eastern Europe, a third cluster could become more clear with eastern populations representing a third eastern refugial group. The difficulty in separating the third cluster could be due to admixture between populations descended from different refugial groups (Petit et al. 2003). Admixture is likely as *S. vulgaris* continues to travel great distances aided by human dispersal, becoming invasive in the United States and other countries. There was widespread occurrence of admixed individuals (0.25 < *Q* < 0.75) in this study, but the results did not differ when removing these individuals from the analysis.

The CCSM model hindcasted suitable habitat on the entire coastline of the Iberian Peninsula, western coast of France, Italy and surrounding islands, southern Greece, north of Greece around Serbia, coastal areas around the Black Sea, and the northern coast of Africa. These areas could all have potentially served as glacial refugia for *S. vulgaris*, making it possible for refugia to exist on all three southern peninsulas as seen in many other species (Taberlet et al. 1998; Hewitt 2004; Schmitt 2007). While there are multiple potential refugial areas for *S. vulgaris* within the Central and eastern European regions based on the SDMs presented here, the genetic data do not allow a determination of the specific number and locations of which of these refugia were likely to be occupied. However, the models suggest locations where *S. vulgaris* could exist based on SDM habitat suitability within the broad regions predicted by the genetic data. Further genetic sampling using more markers could clarify the results and make it possible to match predicted refugia locations with areas of suitable habitat.

Many studies make the assumption that climate variables alone are sufficient for projecting species distributions back into the LGM, as many other environmental predictors are unavailable for this period. However, it is important to check this assumption before projecting LGM distributions, as the LGM prediction would most likely become biased if climate were not the primary environmental driver determining the species' distribution. The model of current *S. vulgaris* distribution showed that climate was by far the primary explanatory factor compared to other land‐based attributes which contributed little predictive value. Therefore, *S. vulgaris'* strong dependence on climate strengthens the findings of the LGM suitability models, and further suggests that *S. vulgaris* tracked suitable climates since the LGM, supporting the postglacial expansion hypothesis.

## Conclusion

Postglacial expansion from glacial refugia since the LGM has been supported for a variety of taxa on many different parts of the planet. Many of these studies use genetic analyses and are beginning to use SDM as a powerful complement. However, one type of species that may not adhere to typical climate‐tracking trends is a widespread weed dispersed by humans and capable of growing in a variety of climates. Weeds of cultivation may have spread primarily by agriculture rather than by postglacial expansion, as was recently found for two *Lolium* species (Balfourier et al. 2000), but not for *Arabidopsis thaliana* (Beck et al. 2008). Our results for the widespread weed *S. vulgaris* support the hypothesis of postglacial expansion from southern refugia in Europe. This finding builds upon previous genetic studies on *S. vulgaris* with the addition of SDM analyses and uses the pairing of SDMs and population genetics to directly test these two competing hypotheses. As with past phylogeographic studies of weedy plants, our results did not show the exact expected pattern of postglacial expansion seen in other species, but still adequately support many aspects of this route, especially when compared with the alternative agriculture expansion. Predictions of refugial locations in the future would benefit from further sampling in Italy and eastern Europe, as well as expanded sampling across the genome. Finally, we found that including data from environmental variables other than climate and the known distribution of ice sheets and permafrost can significantly enhance confidence in the distribution of species during the LGM, and should be considered in future SDM studies.

## Conflict of Interest

None declared.

## Supporting information


**Appendix S1.** Standardized heterozygosity and observed heterozygosity interpolated using the inverse distance weighting method.
**Appendix S2.** Principal component loadings for the climatic and land‐based variables used in the species distribution models.
**Appendix S3.** *Silene vulgaris* climate suitability in Europe during the last glacial maximum based on the MIROC climate scenario.
**Appendix S4.** Current climate suitability model results for *Silene vulgaris* in Europe.
**Appendix S5.** MESS map for the climate‐only SDM showing the extrapolation of variables in the CCSM climate scenario.
