# Supplementary material for: Investigating past range dynamics for a weed of cultivation, Silene vulgaris
Source: Ecol Evol. 2016 Jun 16;6(14):4800–11. doi: 10.1002/ece3.2250 (PMC4979708; doi:10.1002/ece3.2250)

**SUPPORTING INFORMATION**

**Investigating past range dynamics for a weed of cultivation, *Silene vulgaris***

Megan E. Sebasky, Stephen R. Keller, Douglas R. Taylor

**Appendix S1:** Standardized heterozygosity and observed heterozygosity interpolated using the inverse distance weighting method.


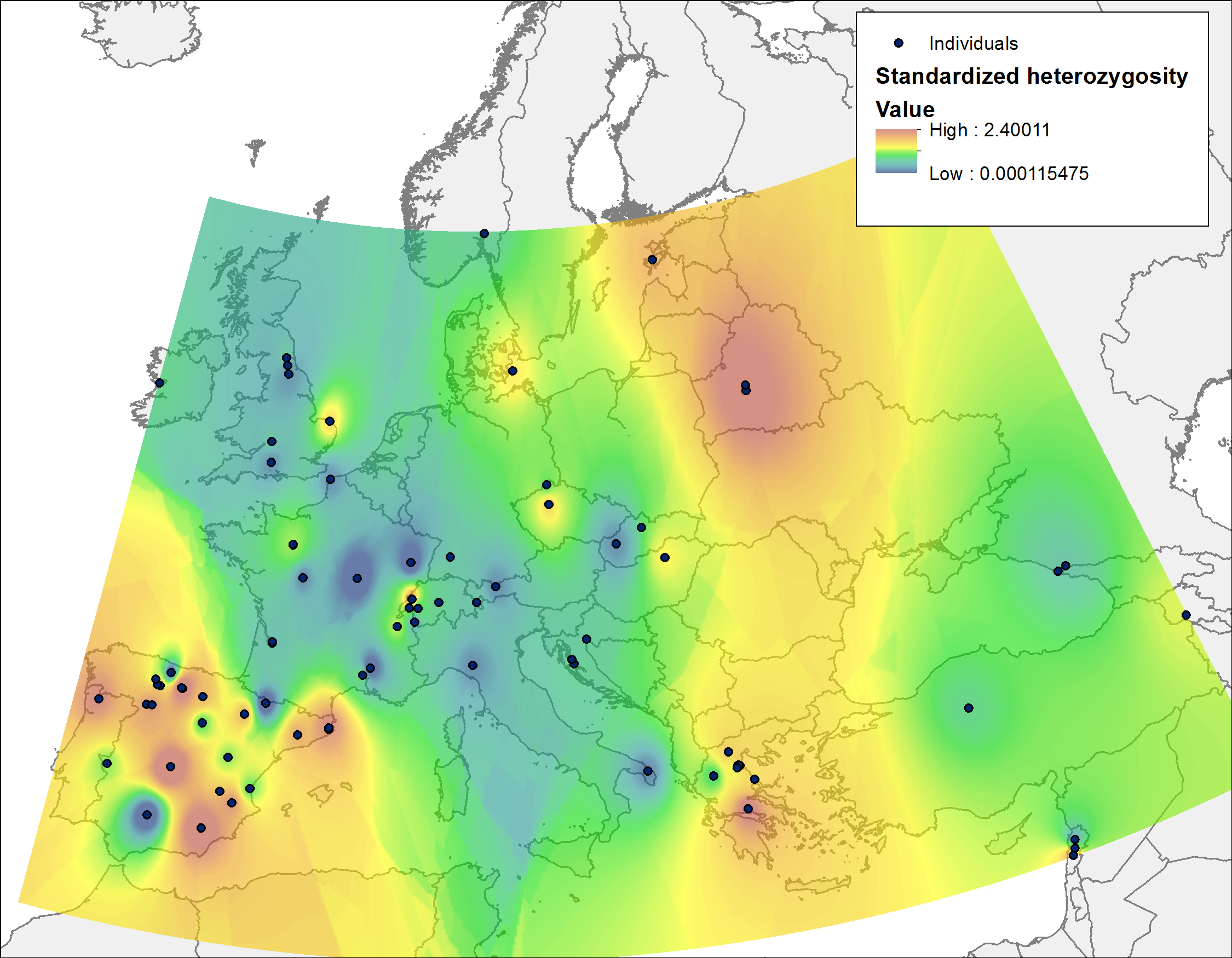


**
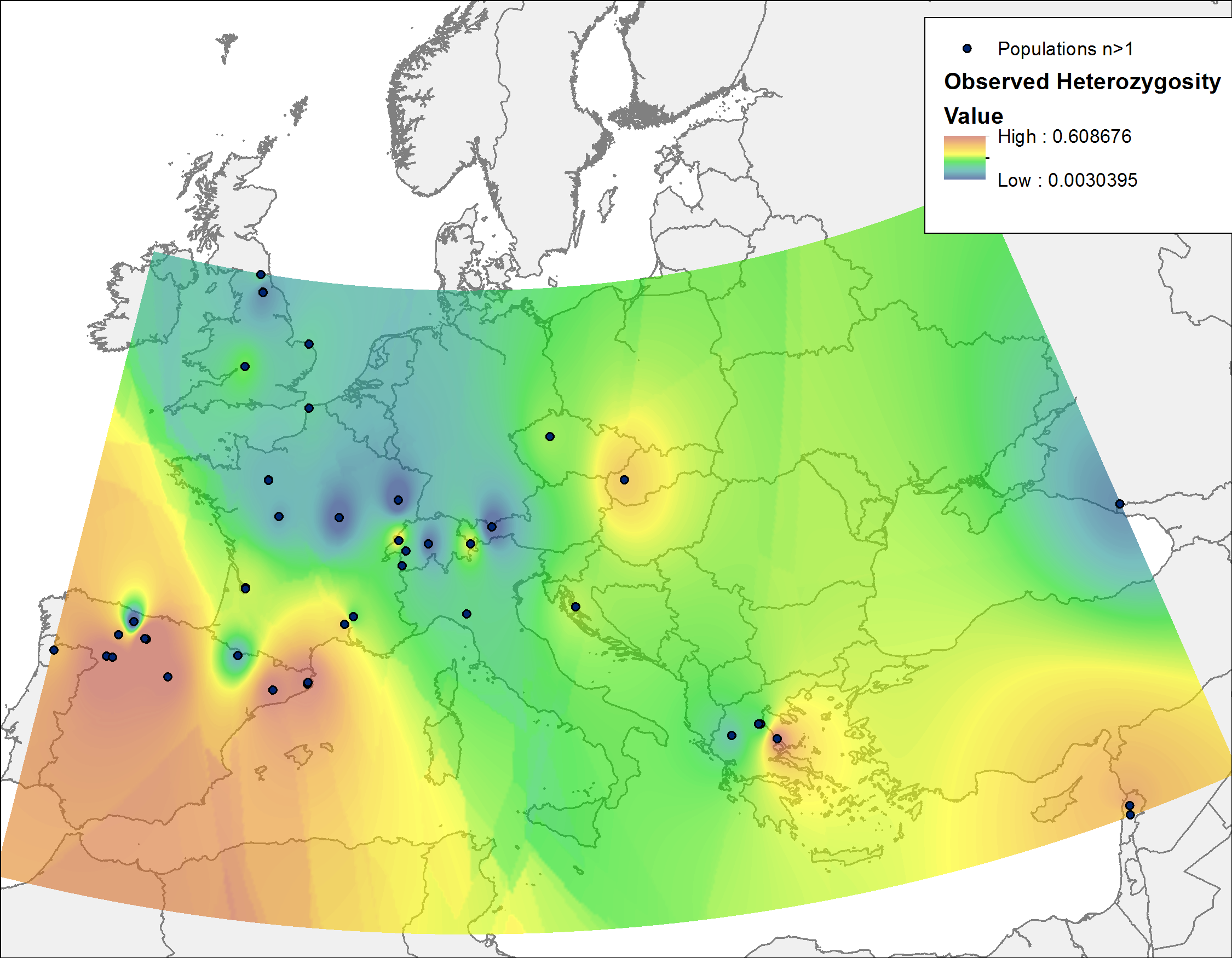
**

**Appendix S2:** Principal component loadings for the 19 Bioclim variables and land-based variables.

| Bioclim variable name | Var. code | Prin1 | Prin2 |
| --- | --- | --- | --- |
| Annual mean temperature | BIO1 | 0.295651 | 0.183803 |
| Mean diurnal range | BIO2 | 0.264726 | -0.079484 |
| Isothermality | BIO3 | 0.226693 | 0.221137 |
| Temperature seasonality | BIO4 | -0.030241 | -0.343061 |
| Max temp warmest month | BIO5 | 0.327054 | 0.017918 |
| Min temp coldest month | BIO6 | 0.218027 | 0.286745 |
| Temperature annual range | BIO7 | 0.076682 | -0.321912 |
| Mean temp wettest quarter | BIO8 | 0.002915 | -0.156092 |
| Mean temp driest quarter | BIO9 | 0.282637 | 0.193623 |
| Mean temp warmest quarter | BIO10 | 0.319052 | 0.061528 |
| Mean temp coldest quarter | BIO11 | 0.249632 | 0.263219 |
| Annual precipitation | BIO12 | -0.208449 | 0.302098 |
| Precipitation wettest month | BIO13 | -0.128839 | 0.293623 |
| Precipitation driest month | BIO14 | -0.276173 | 0.186871 |
| Precipitation seasonality | BIO15 | 0.243416 | -0.01037 |
| Precipitation wettest quarter | BIO16 | -0.138235 | 0.29895 |
| Precipitation driest quarter | BIO17 | -0.267248 | 0.209454 |
| Precipitation warmest quarter | BIO18 | -0.314683 | 0.049543 |
| Precipitation coldest quarter | BIO19 | -0.026353 | 0.352148 |

| Variable name | Prin1 | Prin2 |
| --- | --- | --- |
| Human influence index | 0.03927 | -0.552748 |
| Full soil code from WRB | 0.434563 | 0.024799 |
| Dominant land use | 0.454067 | 0.026748 |
| Dominant parent material | -0.454068 | -0.026778 |
| Land use | -0.04486 | 0.551791 |
| Full soil code (1974 FAO-UNESCO) | 0.43436 | 0.026196 |

**Appendix S3:** Last glacial maximum (LGM) climate suitability for *Silene vulgaris* in Europe based on the Model for Interdisciplinary Research on Climate (MIROC) climate scenario. Extent of the Eurasian ice sheet is sketched based on Svendsen *et al.*, 2004.

*
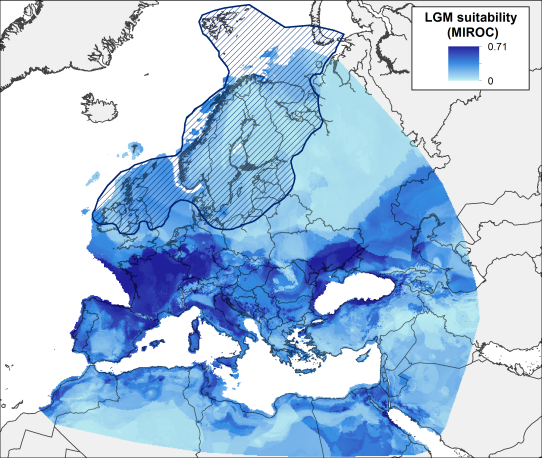
*

**Appendix S4:** Current climate suitability for *Silene vulgaris* in Europe based on a MaxEnt model using (a) land and climate variables and (b) only climate variables. Points shown and used in the model are a combination of edited data from GBIF, BSBI, and lab collections.


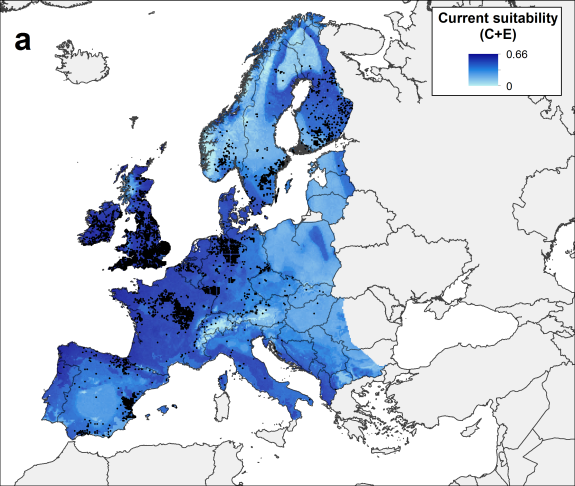


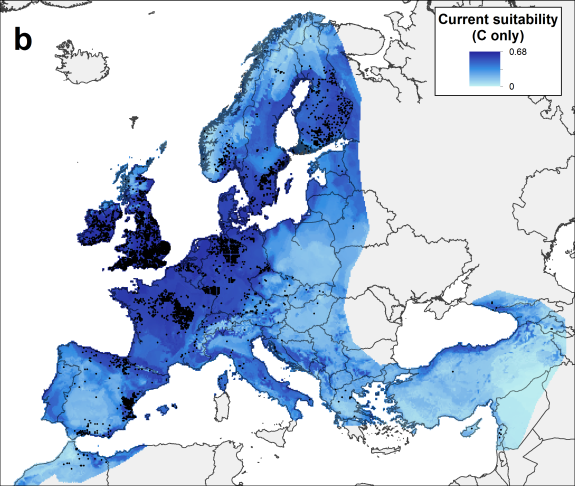


**Appendix S5:** MESS map for the climate-only model, showing where variables were extrapolated in the LGM prediction (CCSM climate scenario).


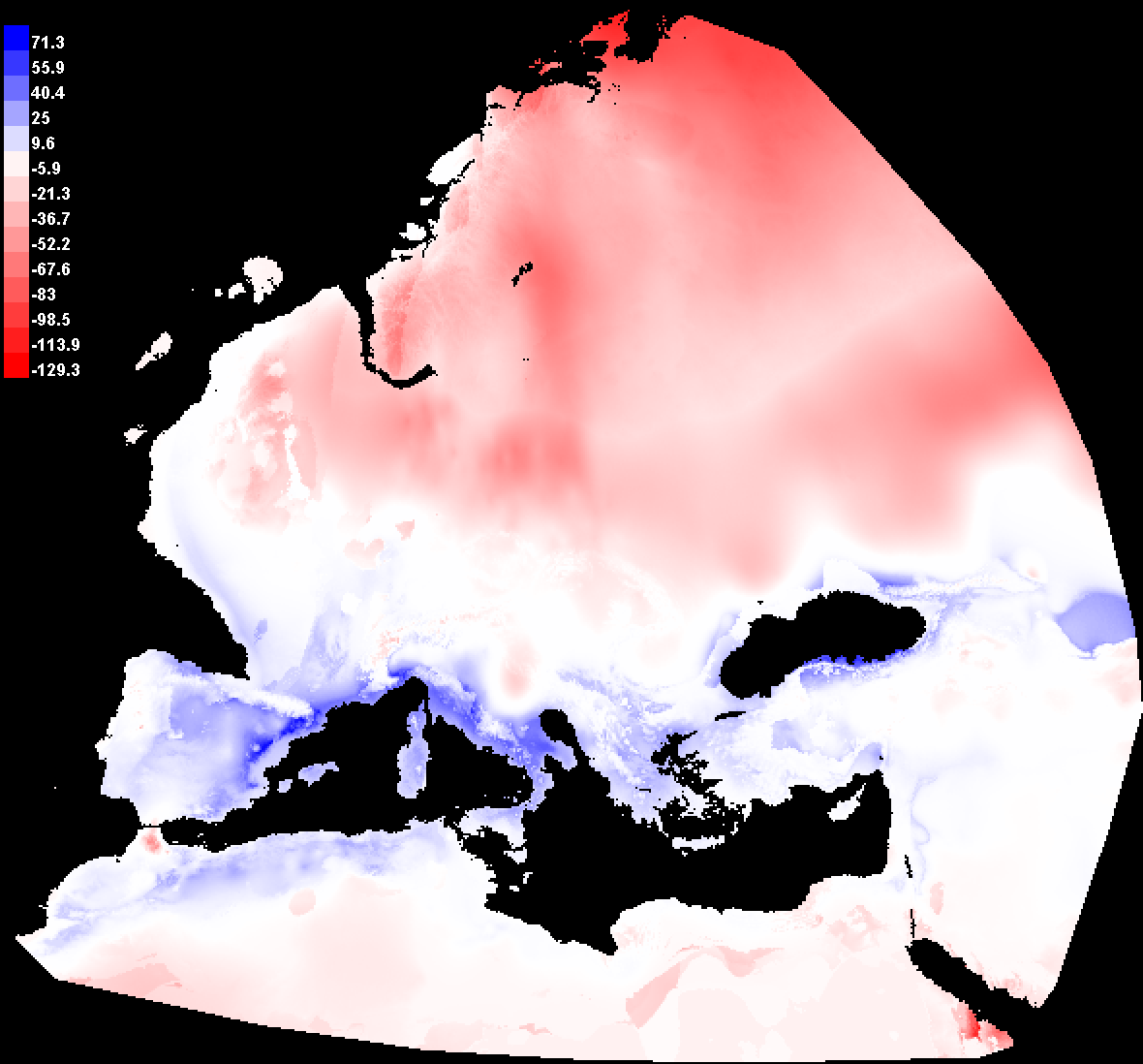

Supplement: Supplementary file 1 — Appendix S1. Standardized heterozygosity and observed heterozygosity interpolated using the inverse distance weighting method. Appendix S2. Principal component loadings for the climatic and land‐based variables used in the species distribution models. Appendix S3. Silene vulgaris climate suitability in Europe during the last glacial maximum based on the MIROC climate scenario. Appendix S4. Current climate suitability model results for Silene vulgaris in Europe. Appendix S5. MESS map for the climate‐only SDM showing the extrapolation of variables in the CCSM climate scenario. [file ECE3-6-4800-s001.docx]
